# „Und das hat mir geholfen, das Darüber-Reden.“: Kindheit in Normalheimen der DDR – Oral History in der (medizinischen?) Praxis

**DOI:** 10.1007/s00048-024-00379-0

**Published:** 2024-02-29

**Authors:** Constanze Reila Schliwa

**Affiliations:** Institut für Historische Bildungsforschung, Universität Hamburg, Von-Melle-Park 8, 20146 Hamburg, Deutschland

## Einleitung

„Bitte erzähle mir deine Lebensgeschichte als ehemaliges ‚Normalheimkind‘^1^.“ Diese Erzählaufforderung bildete den Grundstock der Interviews, die ich für mein Dissertationsprojekt *Erinnern und Verarbeiten des Aufwachsens in Normalkinderheimen der DDR von 1965 bis 1989. Eine Oral History-basierte Untersuchung* führen durfte. Sie thematisch fokussiert zu formulieren, war eine bewusste Entscheidung, werden biografisch-narrative Interviews doch erst dann zu erzählten Lebensgeschichten, wenn die Interviewten selbst bestimmen, von welchen Erinnerungen sie wie ausführlich erzählen (Rosenthal 2015: 50–52). Aus erziehungswissenschaftlicher Sicht mögen die den Lebensgeschichten zugrunde liegenden Lern- und Bildungsgeschichten (Schulze 1993: 176) im Mittelpunkt stehen. Die Erzählaufgabe und Interviewform provozieren aber, dass auch auf Ereignisse außerhalb der Heimzeit eingegangen wird. Diese können für die Auswertung lohnend sein, um bestimmte Handlungs- und Verhaltensweisen vor der Folie des Erlebten zu rekonstruieren oder latentes Wissen sichtbar zu machen (Lucius-Hoene & Deppermann 2004). Darüber hinaus ermöglichen derartige Oral-History(-OH)-Interviews, „neue Fragen aufzuwerfen, die die Forschung bis dato nicht gestellt hat und die sich aus den Antworten der Zeitzeugen ergeben“ (Obertreis 2012: 11). Inwieweit medizinische Fragen in meinem Projekt vertreten sind, sie sich von anderen Forschungszweigen unterscheiden beziehungsweise für diese anschlussfähig sind, wird in diesem Beitrag zu zeigen sein. Vorausgeschickt sei, dass meine Arbeitsschwerpunkte im Bereich der historischen Bildungsforschung, insbesondere der DDR-Heimpädagogik, liegen. Das begründet, weshalb ich vor allem aus einer deutschsprachigen Perspektive schreibe. Gern lade ich auch andere OH-Forschende, die wie ich keine Mediziner:innen oder Medizinhistoriker:innen sind, zu einer kritischen Reflexion des speziellen Status’ der OH in der Medizin ein.

## Biografische Erzählungen im medizinischen Diskurs

Erzählte Lebensgeschichten rekurrieren stets auf subjektive Erinnerungen, weswegen gedächtnistheoretische Fragestellungen berücksichtigt werden müssen. Mit Gedächtnis und Erinnerung setzen sich verschiedene Wissenschaftsdisziplinen unterschiedlich auseinander, unter anderem die Medizin. Folglich rückt ebenso die Biografieforschung immer weiter in das Blickfeld ihrer wissenschaftlichen Untersuchungen. Die Zeitschrift *BIOS* hat sich unter anderem zum Ziel gesetzt, die „Frontstellung zwischen den methodischen Ansätzen mit ihren wissenschaftstheoretischen Traditionen zu überwinden“ (o. A. 2001: 3). 2006 veröffentlichte sie ein Heft zum Thema *Biographische Rekonstruktionen in der Medizin*. In einem der Beiträge diskutiert Klein, inwiefern medizinische Diskurse mit Patient:innen- und Mediziner:innenbiografien verflochten sind und welcher Erzählmuster sich dabei bedient wird. Er stellt unter anderem fest, dass Mediziner:innen und Umwelt einander wechselseitig beeinflussen können – etwa, wenn die Berufswahl durch Schlüsselerlebnisse geprägt wurde. Des Weiteren gebe es strukturelle Ähnlichkeiten zwischen (auto-)biografischen Erzählungen und beispielsweise Anamnesegesprächen. Nicht zuletzt werde die psychologische Arbeit ebenfalls von biografischen Erzählungen getragen (Klein 2006: 8–11).

Ein Vorzug der OH ist, dass sie sich für historische Akteur:innen interessiert, deren Erinnerungen zugunsten von Sichtweisen der oberen Instanzen oder vermeintlicher Stellvertreter:innen marginalisiert worden waren (Wierling 2003: 84). Dies spielt auch in meiner Studie eine Rolle, da die Lebensgeschichten ehemaliger „Normalheimkinder“ bislang seltener erfasst worden sind als von Personen, die in Spezialheimen untergebracht waren. Häufig wird die Auffassung vertreten, die eigene Biografie sei weniger relevant. Das macht es aber umso schwerer, wissenschaftliche Befunde adäquat einzuordnen. Indem nun diese Personen interviewt und ihre Erinnerungen für die Analyse zur Verfügung gestellt werden, können sowohl Perspektivwechsel stattfinden als auch neue Forschungsanreize geliefert werden. Darauf können unterschiedliche Wissenschaftszweige mit ihren spezifischen Methoden reagieren und fachübergreifend daran arbeiten.

## Lebensgeschichten als Krankheitsgeschichten?

Dass Heimerfahrungen Krankheitsgeschichten begünstigen können, konstatieren Sack & Ebbinghaus in ihrem Gutachten für den Expertisenband *Aufarbeitung der Heimerziehung in der DDR* (2012). Anhand der Erzählungen ehemaliger „Heimkinder“ und Einschätzungen von Mitarbeitenden der Beratungsstellen leiten sie drei Kategorien ab. Hierbei muss bedacht werden, dass Aussagen von Personen, die in Normalheimen lebten, abermals unterrepräsentiert sind (ebd.: 314). Dennoch konnte ich bei der Auswertung meines Materials ähnliche Ergebnisse ausmachen: Personen des ersten Typs verfügen über ein negatives Selbstkonzept, neigen eher zu introvertiertem, zum Teil gar depressivem Verhalten (ebd.: 343). Klaus-Peter^2^ erzählt etwa, dass seine Stigmatisierungserfahrungen nicht auf die Zeit im Heim begrenzt geblieben seien, sondern bis ins Erwachsenenalter nachwirken. So habe man ihm während seiner Ausbildung „gesagt, die würden nie wieder einen nehmen, der aus’m Heim […] kommt“. Er macht Flashbacks traumatischer Erlebnisse dafür verantwortlich, dass er unter Alkoholeinfluss einen Unfall mit seinem Motorrad hatte und dabei ein Bein verlor. Nachdem er von einem Psychologen nach der Unfallursache gefragt worden sei, habe dieser abweisend reagiert: „Ach so, Sie waren in Heimunterbringung.“ In seiner Narration assoziiert er die anderen „Heimkinder“ mit Feindseligkeit, Verrat und Gewalt. Infolge dieser Negativerfahrungen und des anhaltenden Gefühls der Stigmatisierung traue er heute „kaum noch jemandem über’n Weg“ und leide unter „Verlustängsten“.

Dem stehen Vertreter:innen des zweiten Typs gegenüber, die als temperamentvoll und offensiv charakterisiert werden. Das führen Sack & Ebbinghaus auf die hierarchischen Strukturen der Heimkollektive zurück, innerhalb derer Positionen zum Teil gewaltsam besetzt und verteidigt werden mussten. Andreas erinnert das Aufwachsen im Normalheim als „sehr entspannt“. Er habe sich nicht „unterordnen“ können und sei „eigentlich immer oben in der Hierarchie“ gewesen. Forderungen seien von ihm und den anderen Jugendlichen an der Spitze zum Teil durch Prügel eingetrieben worden („wenn einer das nicht so gemacht hat wie wir das wollten, gab’s eine“). Im Gegenzug erzählt Andreas von Situationen, in denen die Tages- und Hausordnung ihn in seiner Freiheit begrenzt hätten. Aus diesem Grund könne er heute in keinem Angestelltenverhältnis arbeiten. Wiederholt sei es zu Konfrontationen mit Vorgesetzten gekommen, da er sich „unterdrückt“ gefühlt habe: „Das ist durch den Aufenthalt im Kinderheim wirklich […]. Nee, ich lass mir von keinem mehr was aufdiktieren.“

Personen des dritten Typs setzen sich aktiv mit ihrer Heimzeit auseinander, allerdings aus unterschiedlichen Beweggründen. Während die einen ihre Geschichte aufarbeiten möchten, versuchen die anderen, ihre Erinnerungen durch übersteigerte Unterstützung anderer Betroffener zu verdrängen. Holger, der sich in einem Verein für ehemalige „Heimkinder“ engagiert, erklärt: „Es gibt ja auch viele, die es nicht geschafft haben. Und mein Anliegen war es ja auch, sozusagen in diesem Verein auch an diese Leute zu erinnern.“ Neben dem Austausch mit anderen Ehemaligen organisiere er Veranstaltungen und schreibe Kurzgeschichten und Gedichte. Letzteres verfolge zugleich den Zweck, seine eigenen Kinder an seiner Lebensgeschichte teilhaben zu lassen. Seine Frau zeige sich hingegen desinteressiert. In Bezug auf die Argumentation von Sack & Ebbinghaus könnte Holgers Engagement stärker sein als es von ihm selbst erlebt und wahrgenommen wird. Die Zurückhaltung seiner Frau ist eventuell als Reaktion darauf zu lesen. Im selben Verein wirkt Ingeborg. Anders als von Sack & Ebbinghaus dargestellt, verdrängte sie die Auseinandersetzung mit ihrer Kindheit und Jugend nicht durch die Aufopferung für andere Ehemalige. Dass Ingeborg die Heimzeit „immer nach hinten geschoben“, sie „gar nicht verarbeitet“ habe, sei ihr erst mit dem Auszug ihrer Kinder bewusst geworden. Zudem sei sie beruflich überlastet gewesen. Als bei ihr das Burnout-Syndrom diagnostiziert wurde, habe sich Ingeborg in psychotherapeutische Behandlung begeben.^3^

Der strikte Tagesablauf und die geregelten Strukturen können bei ehemaligen „Heimkindern“ zu entgegengesetzten Extremen geführt haben. So erzählen einige der Interviewten, die Routinen bisweilen in ihren heutigen Alltag übernommen zu haben – zum Beispiel feste Putztage (Ingeborg: „Aber denn hat man ja sonnabends zu Hause geputzt wie ’ne Irre.“) oder die hohe Gewichtung von Pünktlichkeit (Klaus-Peter: „Alles nach Zeit und, ja, musst du alles gleich erledigen.“). Andere lehnen solche tagesstrukturierenden Vorgaben ganz bewusst ab, zum Teil um sich so von ihrem Leben im Heim zu distanzieren (Marina: „Ich habe in meinem Leben so viel geputzt. Ich will nicht mehr.“ Ebd.: 343–344). Zusätzlich zu den psychischen Folgen werden körperliche Beschwerden angesprochen. Diese seien dem Umstand geschuldet, dass Vorsorgeuntersuchungen oder ärztliche Behandlungen in den Heimen nicht oder nicht im angemessenen Maß durchgeführt worden seien. Auch sei den „Heimkindern“ nicht vermittelt worden, sich im Sinne einer Selbstfürsorge um die eigene Gesundheit zu kümmern (ebd.: 345). In den von mir analysierten Interviews werden körperliche Probleme zum Teil auf die Arbeitserziehung zurückgeführt (Simone: „Und ich habe ja immer körperlich gearbeitet. Irgendwann sagt der Körper Nee.“). Offensichtlich fließen in die biografischen Darstellungen ebenso Erinnerungen ein, die für die Entwicklung und Untersuchung spezifischer Krankheitsbilder Gewicht haben können.

## „Weiß ja nicht, wie Sie das so sehen …“ – Methodische und praxeologische Herausforderungen

Lebensgeschichten, die in narrativen Interviews wiedergegeben werden, werden interaktiv hervorgebracht, wobei die Interviewer:innenrolle darauf reduziert ist, aktiv zuzuhören. Demnach weisen offene, qualitative Interviews Parallelen zu (therapeutischen) Beratungsgesprächen – etwa der Psychoanalyse – auf (Kruse 2014: 311) und werden zum Teil auch von den Befragten als solche verkannt. In diesen Fällen wurde mir psychologisches Fachwissen unterstellt, seltener sogar der Wunsch geäußert, im Anschluss an das Interview gemeinsam Coping-Strategien zu erarbeiten. Dies kann problematisch werden, wenn es der interviewenden Person nicht gelingt, ihre Funktion und Arbeitsweise sowie fachspezifischen Fähigkeiten und Grenzen für die Interviewten transparent zu machen. Auch Jureit (1999) zweifelt den therapeutischen Effekt derartiger Interviews an, besonders, da die jeweiligen Rollen der Interviewbeteiligten einer therapeutischen Beziehung entgegenstehen (ebd.: 53). Maindok (2003) vertritt ebenfalls die Position, sozialwissenschaftliche Interviews nicht mit therapeutischen Gesprächen zu verwechseln. Nicht nur liegen beiden Gesprächsarten unterschiedliche Voraussetzungen zugrunde (unter anderem Interesse an subjektiven Relevanzsystemen versus „emotionale Bedürftigkeit des Klienten“), sie setzen auch ihre Schwerpunkte anders (Zugänglichmachen versus „emotionale Heilung“ von Erinnerungen; ebd.: 170–171). Ferner laufen lebensgeschichtliche Interviews Gefahr, retraumatisierend zu sein oder traumatische Erinnerungen, die bislang nicht oder nicht hinreichend verarbeitet worden sind, hervorzurufen. Küsters (2009) empfiehlt, das Interview dann nicht abzubrechen, in der Interviewer:innenrolle zu bleiben und möglichst sachlich auf die Situation zu reagieren (ebd.: 68). Nach dem Interview sollten die Befragten nicht mit ihren Erinnerungen sowie davon ausgelösten Gefühlen und Gedanken allein gelassen, sondern es sollte ihnen angeboten werden, die/den Forschende:n bei Bedarf zu kontaktieren. Ich habe im Rahmen meiner Studie zumeist die Erfahrung machen dürfen, dass das Erzählen der eigenen Lebensgeschichte für die ehemaligen „Heimkinder“ einen entlastenden Effekt hatte. Dies führten sie nicht selten darauf zurück, sich einer fremden Person anvertrauen zu können, ohne dabei ihr Ansehen zu gefährden (ebd.: 49).

Im Gegenzug zu Interviewformen, die einem Frage-Antwort-Muster folgen, lassen biografisch-narrative Interviews „einen umfassenderen und in sich strukturierten Zugang zur Erfahrungswelt“ zu (Flick 2019: 227). Wie oben skizziert, bleiben die Datenerhebung und -auswertung somit nicht ausschließlich auf den Erkenntnisinteressen oder Themen, die der Expertise des/der Forschenden entsprechen, begrenzt. Andere Inhalte, die er/sie zuvor gar nicht bedacht hatte, die deshalb aber nicht minder relevant sind, können so in die Untersuchung aufgenommen werden und Anknüpfungspotenziale für andere Wissenschaftsdisziplinen schaffen. Das bedeutet jedoch auch, dass Länge und Ausgang des Interviews nicht immer vorhersehbar sind. So werden zum Beispiel Schwerpunkte auf die Krankheits- oder, wie bei einem Beispiel aus meinem Material, die Berufsbiografie gelegt, die zwar aus der Heimerfahrung resultieren können, das ursprüngliche Forschungsinteresse aber nur noch ausschnittweise bedienen oder lediglich tangieren. Der Fokus muss in solchen Fällen im Nachfrageteil wieder umgelenkt werden. Das Erinnern an und Sprechen über Situationen und Lebensabschnitte, aus denen die Erzählenden selbstbewusster hervorgegangen sind, kann als individuelle Coping-Strategie fungieren. Mit Blick auf das eben angeführte Beispiel könnte die starke Fokussierung des beruflichen Aufstiegs ebenso den Versuch abbilden, eine „Normalbiografie“ (im Sinne einer Erfolgsgeschichte) zu konstruieren.

In meinem Datensatz sind außerdem lebensgeschichtliche Darstellungen vertreten, die in einem detailarmen Erzählmodus verbleiben. Sie enthalten vorwiegend beschreibende und Belegerzählungen sowie generalisierte Aussagen. Ein Erklärungsansatz könnte darin zu suchen sein, was Mannheim (1980) unter „konjunktiver Erfahrung“ versteht: Das Kollektiv eines Normalheims kann als verbindender Erfahrungsraum interpretiert werden. Seine Angehörigen teilen gemeinsame biografische Erfahrungen, die in kollektives, „atheoretisches“ Wissen (ebd.: 73–76, 211–215) münden. Möglicherweise haben aufgrund von langen Heimunterbringungszeiten Normalitätsverschiebungen stattgefunden. Da mich neben den Lerngeschichten auch die eigenen Auslegungen und Selbstdeutungen der Interviewten interessieren, habe ich versucht, möglichst wenig davon preiszugeben, was ich bereits über die DDR und ihre Heimpädagogik weiß. Dessen ungeachtet wurde mir in meiner Rolle als Wissenschaftlerin ein gewisser Expertinnenstatus – mitunter über meine tatsächlichen Kompetenzen hinaus (etwa hinsichtlich des Kombinats der Sonderheime für Psychodiagnostik und pädagogisch-psychologische Therapie oder der Medikamentengabe in den Heimen) – unterstellt und von gemeinsamen Wissens- und Denkstrukturen ausgegangen.

## Ist OH in der Medizin nun eine Besonderheit?

Medizinische Diskurse können sich in Lebensgeschichten ehemaliger „Normalheimkinder“ niederschlagen. Das umfasst nicht nur körperliche Beschwerden oder psychische Erkrankungen, sondern auch Verhaltensauffälligkeiten, worunter ich die von Sack & Ebbinghaus angeführten „Tics“ verstehe. Ursachen können Stigmatisierung sein (s. Klaus-Peter) oder Handlungsstrategien, die in den Heimen angeeignet und gefestigt worden sind (s. Andreas). Themen, die schon in den Heimkollektiven verhandelt wurden, können – in Ansätzen und auf einem anderen Qualitätsniveau – selbst im Erwachsenenalter wirken. Diese Befunde sind nicht ausschließlich aus medizinischer Perspektive bedeutsam. Sie bedienen zugleich mein eigenes Relevanzsystem, indem sich Aussagen darüber treffen lassen, welche Erinnerungen Normalheime im Allgemeinen generieren und wie die Heimvergangenheit in den gegenwärtigen lebensgeschichtlichen Kontext eingebettet wird. Letzteres lässt wiederum Rückschlüsse auf das Gewordensein der Befragten zu.

Bestätigt werden konnte ferner, dass das Erzählen der eigenen Lebensgeschichte von den Interviewten in der Regel positiv wahrgenommen wird, weil sie eigene Relevanzen setzen (zum Beispiel Folgesymptome der Heimerfahrung) und sich einer fremden Person anvertrauen können, der sie einen gewissen Expert:innenstatus zuschreiben. Einerseits wird OH hier dem Ruf gerecht, den Blick für bisher nicht mitgedachte Themen zu öffnen. Diese können wiederum von unterschiedlichen Disziplinen, darunter die Medizin, aufgegriffen und mit verschiedenen theoretischen Gerüsten untersucht sowie fachübergreifend Unterstützungsangebote für Betroffene entwickelt werden. Auf der anderen Seite sehe ich mich als Erziehungswissenschaftlerin aber auch mit Herausforderungen konfrontiert: Um Lebensgeschichten ehemaliger „Normalheimkinder“ angemessen erheben zu können, muss ich über Hintergrundwissen verfügen. Das ist auch den Befragten bewusst, ohne dies explizit verhandeln zu müssen. Es darf mich nicht überraschen, dass von mir erwartet wird, zeitgenössische Begriffe, Merkmale der Kollektiverziehung oder Ähnliches zu kennen. Anders sieht das bei Fragen zur medizinischen und psychologischen Betreuung ehemaliger „Heimkinder“ in der DDR aus – vor allem, wenn noch nicht hinreichend untersuchte Thesen zur Diskussion gestellt werden. Nicht immer fällt es leicht, in solchen Fällen den eigenen Forschungsschwerpunkt nachvollziehbar eingrenzen, dabei seinen Status aufrechterhalten und dennoch alle wichtigen Informationen erfragen zu können. Rufen das Erinnern und Darüber-Sprechen Retraumatisierungen hervor, gelingt es mir dank meines pädagogischen Backgrounds zwar, adäquat zu handeln. Ich bin mir bestimmter Themen bewusst, erkenne Verhaltensmuster und Coping-Strategien und weiß, wie ich diese bei der späteren Auswertung zu deuten habe. Allerdings kann ich den Interviewten, zumindest in medizinischer Hinsicht, keinerlei Hilfe geben. Es bleibt allenfalls bei einem informellen Austausch im Anschluss an das Interview. Allein das Wissen um das Risiko einer Retraumatisierung kann sowohl die Vorbereitung als auch die Durchführung des Interviews sowie die Haltung der/des Forschenden beeinflussen – unabhängig von ihrer/seiner wissenschaftlichen Schule. Selbst die Auswertung des Datenmaterials bleibt davon nicht unberührt. Möglicherweise wurde die Situation an einigen Stellen stärker von dem/der Interviewer:in beeinflusst als gewollt – etwa, um Befragte (vermeintlich?) zu schützen.

Welchen Mehrwert können Forschungsergebnisse aus der Geschichts- und Erziehungswissenschaft nun für die Medizin haben? Sicher ist diesbezüglich Klein zuzustimmen, wenn er sagt, dass „im reflektierten Umgang mit biographischen Erzählungen […] gerade im medizinischen Kontext große Erkenntnispotenziale [liegen]“ (2006, 13).

## Footnotes

1 Angesichts der stigmatisierenden Funktion des Begriffs setze ich „Heimkind“ in Anführungszeichen.

2 Zum Schutz der Interviewten wurden die Namen und biografischen Daten anonymisiert.

3 Im Rahmen des Interviews schildert die Befragte den Ablauf und die Methoden der Einzel- und Gruppentherapieeinheiten. Das liefert sicher einen weiteren spannenden Ansatz für die Auseinandersetzung mit der Verwobenheit von OH und Medizin, kann aus Kapazitätsgründen und wegen meines Mangels an fachspezifischem Wissen hier aber nicht weiter ausgeführt werden.
